# Metronomic Four-Drug Regimen Has Anti-tumor Activity in Pediatric Low-Grade Glioma; The Results of a Phase II Clinical Trial

**DOI:** 10.3389/fphar.2018.00950

**Published:** 2018-09-27

**Authors:** Arnauld Verschuur, Marie-Amélie Heng-Maillard, Philippe Dory-Lautrec, Romain Truillet, Elisabeth Jouve, Pascal Chastagner, Pierre Leblond, Isabelle Aerts, Stéphane Honoré, Natasha Entz-Werle, Nicolas Sirvent, Jean-Claude Gentet, Nadège Corradini, Nicolas André

**Affiliations:** ^1^Department of Pediatric Oncology, La Timone Children’s Hospital, Assistance Publique Hôpitaux de Marseille, Marseille, France; ^2^Department of Neuroradiology, La Timone Hospital, Assistance Publique Hôpitaux de Marseille, Marseille, France; ^3^CIC-CPCET, La Timone Hospital, Assistance Publique Hôpitaux de Marseille, Marseille, France; ^4^Department of Pediatric Oncology, Children’s Hospital, Nancy, France; ^5^Pediatric Oncology Unit, Oscar Lambret Centre, Lille, France; ^6^Pediatric Department, Institut Curie, Paris, France; ^7^Department of Clinical Pharmacy, La Timone Hospital, Assistance Publique Hôpitaux de Marseille, Marseille, France; ^8^Pédiatrie Onco-Hématologie, CHU Hautepierre, Strasbourg, France; ^9^Department of Pediatric and Adolescent Hematology-Oncology, Children’s Hospital Arnaud de Villeneuve, Montpellier, France; ^10^Department of Pediatric and Adolescent Hematology-Oncology, Hôpital Mère-Enfant, Nantes, France; ^11^Institut National de la Santé et de la Recherche Médicale, Centre National de la Recherche Scientifique, Institut Paoli Calmettes, Centre de Recherche en Cancérologie de Marseille, Aix-Marseille Université, Marseille, France; ^12^Metronomics Global Health Initiative, Marseille, France

**Keywords:** metronomic chemotherapy, pediatric oncology, angiogenesis, low grade glioma, immunity, drug repositioning

## Abstract

**Background:** Metronomic chemotherapy (MC) is defined as the frequent administration of chemotherapy at doses below the maximal tolerated dose and with no prolonged drug-free break. MC has shown its efficacy in adult tumor types such as breast and ovarian cancer and has to some extent been studied in pediatrics.

**Objective:** To assess the anti-tumor activity and toxicity of a four-drug metronomic regimen in relapsing/refractory pediatric brain tumors (BT) with progression-free survival (PFS) after two cycles as primary endpoint.

**Methods:** Patients ≥4 to 25 years of age were included with progressing BT. Treatment consisted of an 8-week cycle of celecoxib, vinblastine, and cyclophosphamide alternating with methotrexate. Kepner and Chang two-steps model was used with 10 patients in the first stage. If stabilization was observed in ≥2 patients, 8 additional patients were recruited. Assessment was according WHO criteria with central radiology review.

**Results:** Twenty-nine patients (27 evaluable) were included in two groups: ependymoma (group 1, *N* = 8), and miscellaneous BT (group 2): 3 medulloblastoma (MB), 5 high grade glioma (HGG), 11 low grade glioma (LGG), 2 other BT. After first stage, recruitment for ependymoma was closed [one patient had stable disease (SD) for 4 months]. Cohort 2 was opened for second stage since 1 HGG and 3 LGG patients had SD after two cycles. Recruitment was limited to LGG for the second stage and 2 partial responses (PR), 6 SD and 2 progressive disease (PD) were observed after two cycles. Of these patients with LGG, median age was 10 years, nine patients received vinblastine previously. Median number of cycles was 6.8 (range: 1–12). Treatment was interrupted in five patients for grade 3/4 toxicity.

**Conclusion:** This regimen is active in patients with LGG, even if patients had previously received vinblastine. Toxicity is acceptable.

**Trial Registration:** This study was registered under clinicaltrials.gov – NCT01285817; EUDRACT nr: 2010-021792-81.

## Introduction

Despite intensive multi-modality treatment, children with brain tumors have a variable prognosis, depending on tumor type, quality of resection, limitations to apply radiotherapy and resistance to chemotherapy. Most patients with relapsed brain tumors have a dismal prognosis. Patients with low grade glioma (LGG) may respond or stabilize to several lines of chemotherapy and vinblastine has proven its efficacy (Bouffet et al., 2012; Lassaletta et al., 2016). However, notwithstanding the “benign” histology of LGG some refractory patients have a poor functional and/or vital long term prognosis (Colin et al., 2013).

Angiogenesis is defined by the development of neovascularization in and around solid tumors supplying nutrients and oxygen to growing tumors. Angiogenesis plays a key role in both tumor growth and the potential metastatic development (Bergers and Benjamin, 2003; Ferrara and Kerbel, 2005; Kerbel, 2008). Anti-angiogenic treatments are now established strategies to treat cancer (Colleoni et al., 2002; Kerbel and Kamen, 2004; Kieran et al., 2005; Sterba et al., 2006; Pasquier et al., 2007, 2010; André et al., 2008; André and Pasquier, 2009). Metronomic chemotherapy (MC) is defined as the frequent administration of chemotherapy at doses below the maximal tolerated dose and without prolonged drug-free intervals (André and Pasquier, 2009; Pasquier et al., 2010; André et al., 2013). Metronomic therapy has a distinct mode of action as compared to maximum tolerated dose (MTD) chemotherapy as used in standard regimens, since it may act through various mechanisms: by assuring continuous exposure during cell cycle, thereby preventing tumor cell regrowth (Kerbel and Kamen, 2004; André and Pasquier, 2009; Pasquier et al., 2010); by causing anti-angiogenic effects through affecting the tumor endothelial cells and by targeting circulating endothelial progenitor cells (CEP) (Ferrara and Kerbel, 2005; Pasquier et al., 2007; Kerbel, 2008; André and Pasquier, 2009), thereby contributing to tumor dormancy; by increasing *in situ* drug delivery to the tumor cells by decreasing the interstitial fluid pressure (IFP) of the tumor environment (Pasquier et al., 2010); by restoring the anticancer activity of the immune system by decreasing regulatory T-cells (T-regs), or inducing dendritic cell maturation (André and Pasquier, 2009; Pasquier et al., 2010) and by direct impact on cancer cells or cancer stem cells (André et al., 2013, André et al., 2017).

As a consequence MC is now regarded as a form of intrinsic multi-targeted chemotherapy.

Metronomic chemotherapy has also been reported to induce less severe adverse events (AEs) usually associated with chemotherapy (Choi et al., 2008; Barber et al., 2013). MC is frequently combined with drug repositioning or repurposing (DR) which consists in reusing old drugs for new indications (André et al., 2013). The combination of MC and DR has been defined as metronomics (André et al., 2013).

Metronomic chemotherapy has shown its efficacy in adult tumor types such as breast and ovarian cancer (Colleoni et al., 2002; Barber et al., 2013). The combination of anti-angiogenic compounds such as bevacizumab with low-dose (LD) cyclophosphamide has shown response rates as high as 42% in refractory ovarian cancer (Barber et al., 2013).

Metronomic therapy has to some extent been studied in pediatrics with a majority of retrospective series and phase I or phase II trials, although randomized trials have also been reported recently (Pramanik et al., 2017; Senerchia et al., 2017). Combinations of thalidomide/celecoxib with alternating LD etoposide/cyclophosphamide (Kieran et al., 2005) showed prolonged disease stabilizations in especially CNS-tumors. Similarly a combination of celecoxib with alternating LD etoposide/LD-cyclophosphamide given as a maintenance lead to sustained disease stabilization in different tumor types (Choi et al., 2008). Continuous celecoxib and on/off isotretinoin with alternating LD etoposide and LD temozolomide (Sterba et al., 2006) may be beneficial in intra- and extra-cranial tumors. Vinca-alcaloids like vinblastine may have anti-angiogenic effects (Pasquier et al., 2007; Senerchia et al., 2017). Vinblastine has shown its anti-tumor efficacy for the treatment of LGG (Bouffet et al., 2012; Lassaletta et al., 2016) and was integrated in metronomic regimens (Heng et al., 2016), where the pharmacokinetics of combinations of vinblastine, cyclophosphamide, and celecoxib were previously studied. However, little is known on the anti-tumor efficacy of this combination.

We previously reported our pilot study with a four-drug metronomic regimen in relapsed/refractory pediatric malignancies (André et al., 2011). This pilot led us to a slightly modified treatment scheme with an 8 week cycle using celecoxib, vinblastine, alternating oral cyclophosphamide, and oral methotrexate. This MC scheme was used for a phase II clinical trial in CNS- and non-CNS tumors. We report here the results for the CNS tumors. The results of the other non-CNS tumors will be reported separately.

## Patients and Methods

Patients could be included in this multi-center, combination phase II, open-label, non-comparative, non-randomized trial. Thirteen pediatric oncology centers pertaining to the French National Pediatric Oncology Society (SFCE) participated in this phase II trial. Approval was obtained from our University Ethical Review Board (CPP Sud Méditerranée I) and national Regulatory Authorities [Agence Nationale de Sécurité du Medicament (ANSM). Consent procedure was according to GCP-guidelines with an age-adapted information and assent form and an information and consent form for parents/guardians (in case of minor patients).

Patients’ age was between 4 and 25 years of age and Lansky-Play Scale should be ≥70% or ECOG Performance status ≤1.

Patients had a histologically or cytologically confirmed malignant solid tumor (DIPG and optic pathway glioma excluded). All progressive or recurrent solid tumors could be included in this Phase II study, provided there were no curative options anymore. There was no maximum in previous lines of therapy; patients that were previously treated by vinblastine were eligible.

Standard adequate hematological and biochemical function were required:

– Absolute neutrophil count ≥ 1.0 × 109/l
– Platelets > 75 × 109/l
– Hemoglobin > 7 g/dl
– Hepatic function: AST/ALT ≤ 3 × ULN and bilirubin ≤ 2 ULN.
– Creatinine > 1.5 × ULN.

Written informed consent of parent/guardian and patient assent was collected before enrollment.

**FIGURE 1:**
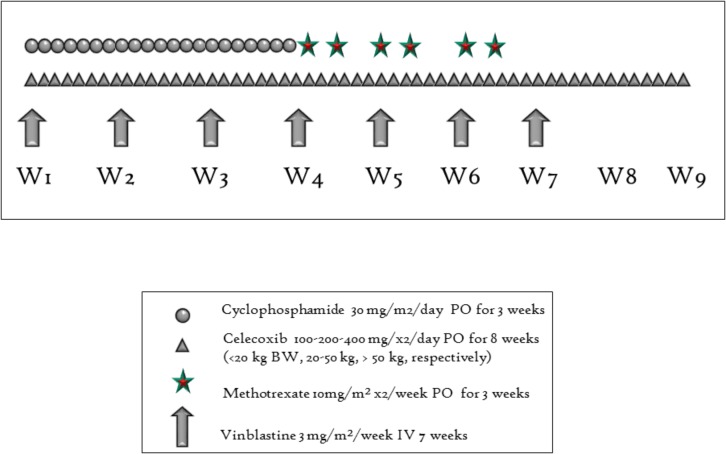
One cycle of treatment consisting of 8 weeks with four compounds.

Treatment consisted of weekly vinblastine 3 mg/m^2^ (weeks 1 to 7), daily cyclophosphamide 30 mg/m^2^ (days 1–21), twice weekly methotrexate 10 mg/m^2^ (days 21–42), and twice daily celecoxib 100–200–400 mg (<20 kg BW, 20–50 kg, >50 kg, respectively), followed by a 13 days chemotherapy break (**Figure 1**).

The primary endpoint was anti-tumor efficacy as defined by progression-free survival (PFS) after two cycles (=8 weeks) as assessed by conventional imaging (CT/MRI) and according to WHO response criteria (World Health Organization, 1979).

Progressive disease (PD): 25% increase in tumor size, or appearance of new lesions; partial response (PR): at least 50% decrease in tumor size; complete response (CR): disappearance of all known lesions; stable disease (SD): neither partial response nor progression.

The secondary endpoints were:

– Progression-free survival and overall survival (OS) after 12 months of treatment.
– Response rate after any number of cycles (best response). Central radiology review was mandatory (PD-L).
– Safety: toxicity according to NCI-CTC v3.

Adverse events were determined through laboratory analyses and investigator observations and classified according to NCI-CTC version 3.0.

Treatment was interrupted in case of:

Grade 3 or grade 4 non-hematological toxicity.
Grade 3 neutropenia or thrombocytopenia for more than 7 days
Grade 3 febrile neutropenia with documented infection.
Grade 4 neutropenia or thrombocytopenia.

### Statistical Considerations and Feasibility

A total of 18 patients were anticipated for accrual according to Kepner and Chang two steps model. Thus, after inclusion of the first 10 patients, if primary objective was reached in less than 2/10 patients the cohort was closed. If primary objective was reached in two or more patients, 8 additional patients were to be recruited. After this second stage if primary objective was reached in overall 6 patients, we concluded that the treatment is efficacious.

Ten patients were to be included in the first stage for ependymoma (group 1) and other malignant brain tumors (including LGG, group 2).

According to the two-step design, we defined that this regimen is efficacious if PFS is over 34% and we considered 10% as the limit under which this treatment is not efficacious. If alpha is 10% and beta 10%, a maximum of 18 patients would be recruited.

Statistical analyses were done on all included subjects without any major protocol deviations who were evaluable for primary outcome, provided they had more than 15 days treatment. Data are summarized for categorical variables as number of patient (%) and continuous variables as median (range). All analyses were summarized using descriptive statistics. Time-to-event analysis were estimated using the Kaplan–Meier method. PFS was defined as the time from the first treatment dose until disease progression and global survival was defined as the time from the first treatment dose until death from any cause. Patients who drop out for any reason other than disease progression or death were censored using the last contact date (we put an upper limit to 730 days). Data management and statistical analysis were done using SAS version 9.4 (SAS Institute, Cary, NC, United States).

clinicaltrials.gov – NCT01285817; EUDRACT nr : 2010-021792-81.

## Results

### Patients’ Characteristics, Accrual, and Study Duration

**FIGURE 2:**
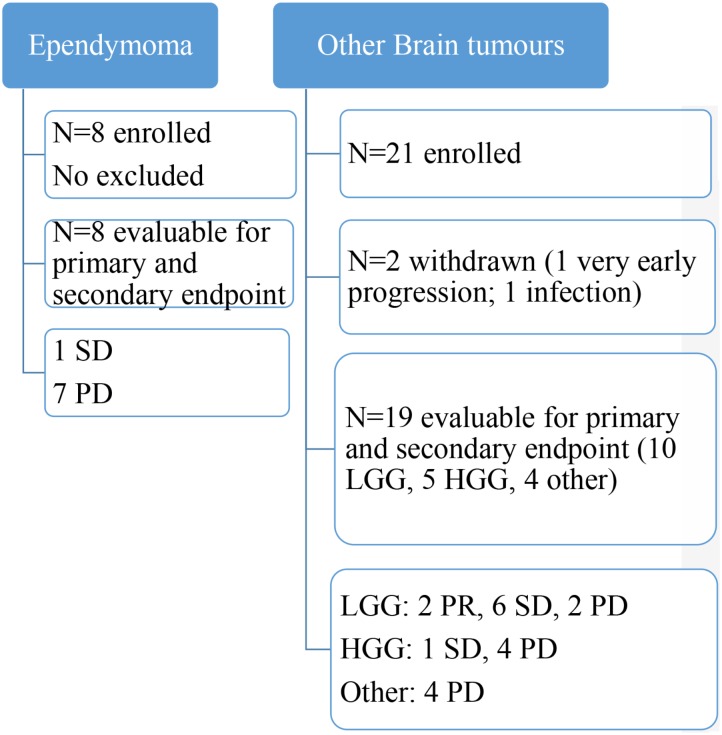
Flowchart of patients’ accrual and assessment. LGG, low grade glioma; HGG, high grade glioma; PR, partial response; SD, stable disease; PD, progressive disease.

Enrolment lasted from January 2011 until March 2015. During this period 29 patients were included in two groups of intention to treat patients: 8 patients with ependymoma and 21 with other BT. The first stage for ependymoma was not completed because of poor enrolment. Central radiology review was completed in November 2016, and the last follow-up analysis was conducted in March 2017. Twenty-seven patients could be assessed after two cycles of MC (4 months). There were 15 boys and 12 girls. A flowchart of enrolled patients is depicted in **Figure 2**.

Median age of these 27 patients was 8.5 years for the ependymoma group and 10 for other BT. Median number of relapses was 2.0 for the ependymoma and 3.0 for the other BT. Fifty percent of ependymoma patients had at least two lines of chemotherapy before inclusion whereas it was ≥3 lines in 58% of other BT (70% for LGG). Details of the entire population and outcomes are reported in **Table 1** (characteristics) and **Table 2** (treatment and outcome).

**Table 1:** Patients’ characteristics and previous treatment and status of disease.

		Other malignant CNS tumor			
	Ependymoma	High grade glioma	Low grade glioma	Other	Total
	(*n* = 8)	(*n* = 5)	(*n* = 10^∗^)	(*n* = 4)	(*n* = 19)
Age at inclusion	8.5 [5–13]	7.5 [4–20]	9.3 [4–15]	13.0 [11–18]	10.0 [4–20]
Sex (% of female)	1 (13%)	1 (20%)	7 (70%)	3 (75%)	11 (58%)
NF-1 status			1 (10%)		1 (5%)
Patients with metastases				1 (25%)	1 (5%)
Anterior surgery or radiotherapy
Only surgery	4 (50%)	1 (20%)	8 (80%)	2 (50%)	11 (58%)
Only radiotherapy		3 (60%)			3 (16%)
Surgery & radiotherapy	4 (50%)	1 (20%)	2 (20%)	2 (50%)	5 (26%)
Nr of previous lines of chemotherapy
0	2 (25%)
1	2 (25%)	3 (60%)		1 (25%)	4 (21%)
2	1 (13%)	1 (20%)	3 (30%)		4 (21%)
3	1 (13%)		1 (10%)		1 (5%)
>3	2 (25%)	1 (20%)	6 (60%)	3 (75%)	10 (53%)
Patients with previous vinca alkaloid			9 (90%)	2 (50%)	11 (58%)

*Data are median [range] and n(%) of patients. Nr, Number. ^∗^Eleven patients with LGG included with one patient not assessable at two cycles*.

**Table 2:** Patients’ treatment and outcome according to histology (Per Protocol population *n* = 27).

		Other malignant CNS tumor			
	Ependymoma (*n* = 8)	High grade glioma (*n* = 5)	Low grade glioma (*n* = 10^∗^)	Other (*n* = 4)	Total (*n* = 19)
Tumor status					
Refractory tumors		3 (60%)	5 (50%)	1 (25%)	9 (47%)
Relapse	5 (63%)	1 (20%)		1 (25%)	2 (11%)
Refractory relapse	3 (37%)	1 (20%)	5 (50%)	2 (50%)	8 (42%)
Number of cycle of treatment received	1.8 [1.3–2.0]	0.5 [0.5–1.5]	6.8 [2.0–11.0]	1.0 [0.8–1.3]	2.0 [0.5–9.0]
Response after 2 cycles of treatment					
Without progression	1 (13%)	1 (20%)	8 (80%)		9 (48%)
Progression	7 (87%)	4 (80%)	2 (20%)	4 (100%)	10 (52%)
“Best response” after any nr of cycle					
CR or PR			2 (20%)		2 (10%)
Stable	1 (13%)	1 (20%)	6 (60%)		7 (37%)
Progression	7 (87%)	4 (80%)	2 (20%)	4 (100%)	10 (53%)
Median of PFS (days)	109	48	–	70.0	105

*Data are median [range] and n(%) of patients. nr, number; PFS, progression-free survival. Median PFS was not attained for LGG. ^∗^Eleven patients with LGG included with one patient not assessable at two cycles*.

### Tumor Assessment and Treatment Duration

One patient with progressive ependymoma had SD for 4 months. The other seven patients with ependymoma showed tumor progression before or at the evaluation after two cycles. For these 8 patients median number of cycles of MC was 1.8 (0.5–3.5). Median PFS was 109 days. This cohort was therefore not open for second stage due to lack of efficacy.

In the cohort of other BT 12 patients were enrolled initially since 2 patients were withdrawn because of early progression (within 1 week; *N* = 1) or osteomyelitis (not-diagnosed as such at inclusion). For this cohort, as per protocol, accrual was opened for second stage since 1 HGG and 3 LGG patients had SD after two cycles. The Independent Data Monitoring Committee and the trial methodologist recommended that recruitment should be limited to LGG for the second stage with one supplementary patient being included. As such 21 patients were enrolled:

– 3 medulloblastoma (MB);
– 5 high grade glioma (HGG) (2 of which DIPG);
– 11 low grade glioma (LGG);
– 2 others.

Overall, 19 “per protocol” patients could be assessed for efficacy at cycle 2. Median PFS was 105 days. One patient with HGG (anaplastic oligodendroglioma) stabilized for 2 years while none of the other HGG, DIPG, or MB stabilized.

**FIGURE 3:**
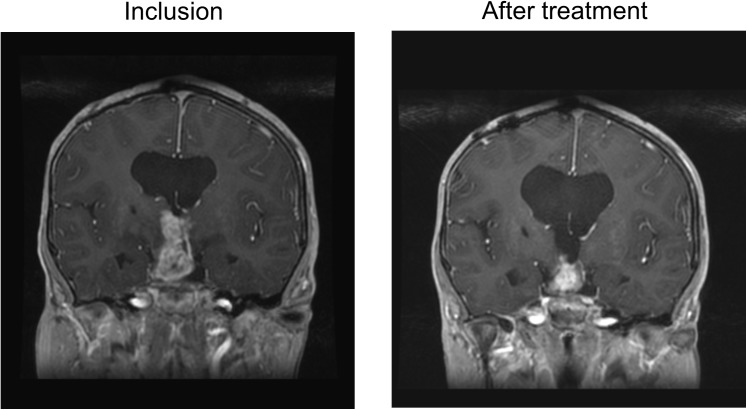
MRI of a patient with LGG at inclusion and after 4 months of MC.

Of the 11 patients with LGG, 10 were assessable for efficacy. Median age was 9.3 years, median duration of illness at inclusion was 6.5 years, median number of relapses 3.5. Nine patients previously received vinblastine, with a median duration of previous vinblastine treatment of 4.4 months while the median interval to previous end of vinblastine treatment was 2.5 years. One patient had neurofibromatosis type I. BRAF status was unknown for these patients at inclusion. Two PR were observed, one after four cycles of MC (**Figure 3**), one after six cycles.

**FIGURE 4:**
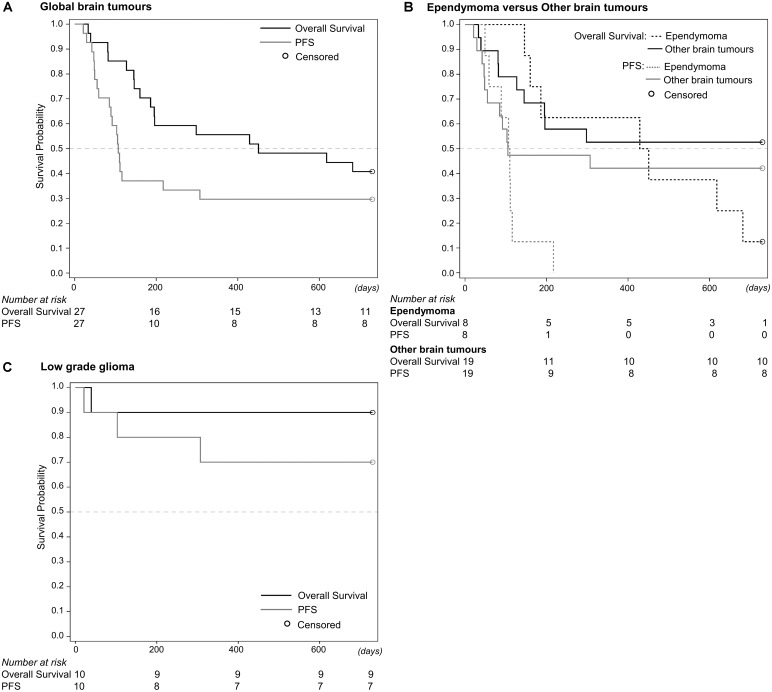
Kaplan–Meier curves for overall survival (OS) and progression-free survival (PFS) of all patients with brain tumors **(A)**, ependymoma and other brain tumors **(B)**, and low grade glioma separately **(C)**.

Among the other patients 6 SD, and 2 PD were observed while one patient was not evaluable after two cycles (**Table 2**). Event-free survival at 1 and 2 years was 70% (**Figure 4**), while OS was 90% at 1 and 2 years. Median number of cycles was 6.8 (range: 1–12). Seven patients received at least 1 year of therapy. Nine patients responded to the primary endpoint of having at least disease stabilization after two cycles (4 months) of therapy. As a consequence as per protocol we concluded that this metronomic regimen is efficacious in the cohort of other BT, especially for LGG.

### Dose Intensity and Adverse Events

The main toxicity was hematologic and among hematologic toxicity, neutropenia was the most common (*n* = 11 patients). Grade 3/4 neutropenia occurred 21 times in 11 patients, with 4 patients (1 HGG, 3 LGG) having had 1 episode of grade 4 neutropenia. Two patients (1 ependymoma, 1 LGG) had grade 4 febrile neutropenia. Grade 3/4 lymphopenia occurred eight times in 5 patients, with only 1 patient with grade 4. No grade 3/4 thrombocytopenia was observed. No grade 4 non-hematological adverse-events were observed. Hepatic enzyme increase was the most common non-hematologic AE (*n* = 8 grade 2/3). Grade 2/3 mucositis occurred in 3 patients. The other non-hematological AEs consisted of grade 2 rhinitis/pharyngitis, fatigue, keratitis/conjunctivitis, diarrhea, anal fissure, paraesthesia, dizziness and grade 3 hypophosphatemia and constipation. Toxicity details are depicted in **Table 3**.

**Table 3:** 2–4 adverse events as observed in the intention to treat population, *n* = 29.

				Other CNS tumors			
	Adverse event	Ependymoma (*n* = 8)	High grade glioma (*n* = 5)	Low grade glioma (*n* = 11)	Other (*n* = 5)	Total (*n* = 21)
Hematologic	**Neutropenia**	**6 (75%)**	**2 (40%)**	**5 (46%)**		**7 (33%)**
	Grade 3	5 (63%)		2 (18%)		2 (10%)
	Grade 4		1 (20%)	3 (27%)		4 (19%)
	**Lymphopenia**	**3 (38%)**	**3 (60%)**	**5 (46%)**	**1 (20%)**	**9 (43%)**
	Grade 3	2 (25%)	1 (20%)	1 (9%)		2 (10%)
	Grade 4			1 (9%)		1 (5%)
	**Anaemia**	**4 (50%)**	**1 (20%)**	**4 (36%)**		**5 (24%)**
	Grade 3		1 (20%)	2 (18%)		3 (14%)
	**Febrile neutropenia**	**1 (13%)**		**1 (9%)**		**1 (5%)**
	Grade 4	1 (13%)		1 (9%)		1 (5%)
Non-hematologic	**ALAT/ASAT**		**1 (20%)**	**5 (46%)**	**2 (40%)**	**8 (38%)**
	Grade 3		1 (20%)	4 (36%)	2 (40%)	7 (33%)
	**Mucositis**	**1 (13%)**		**2 (18%)**		**2 (10%)**
	Grade 3			1 (9%)		1 (4%)
	**Diarrhea**		**1 (20%)**	**1 (9%)**	**1 (20%)**	**3 (14%)**
	**Other AE**	**1 (13%)**	**2 (40%)**	**3 (27%)**	**2 (40%)**	**7 (33%)**
	Grade 3			1 (9%)	1 (20%)	2 (10%)

*Data reflect n(%) of patients, referring to the predominantly observed AE (include grade 2–4). The bolded values are the cumulative frequency of grade 2–4 toxicity (as mentioned in the legend)*.

Treatment was interrupted temporarily in 5 patients for grade 3/4 toxicity (hepatic and/or hematological) and led to temporary dose reductions.

The mean dose intensity of vinblastine (mean amount of drug received and calculated over multiple cycles as a proportion to the per protocol dose) for the 10 evaluable patients with LGG was 90.2% after six cycles (range: 57–100%) and 92.7% after 12 cycles (range: 75–100). One patient had dose interruption and reduction during the first two cycles to 50% of the intended dose because of febrile neutropenia. The dose could ultimately be increased to 100% until the 12^th^ cycle. One patient had only three administrations suspended because of herpes infection and cystitis. All the other patients had 100% of mean dose intensity. The mean dose intensity of cyclophosphamide for these LGG patients was 98.9% after six cycles (range: 93–100) and 99.3% after 12 cycles (range: 96–100). The mean dose intensity of methotrexate for the same 10 evaluable patients with LGG was 93% after six cycles (range: 76–100) and 93% after 12 cycles (range: 69–100) and for celecoxib the mean dose intensity was 92.0% after six cycles (range: 67–100) and 95% after 12 cycles (range: 81–100).

## Discussion

We report here the results for the two groups of children with relapsing or refractory brain tumors included in a multicentre phase II clinical trial using a four-drug metronomic regimen.

We developed this new multidrug metronomic regimen for children with brain tumors and extra-cerebral tumors, integrating the different mechanisms of action of MC. Indeed, although MC was initially considered to be an anti-angiogenic therapy (Bergers and Benjamin, 2003; Kerbel and Kamen, 2004; Ferrara and Kerbel, 2005; Kerbel, 2008), recent findings have highlighted new effects, which all likely contribute to treatment efficacy (Pasquier et al., 2007, 2010; André and Pasquier, 2009; André et al., 2013, 2017). These effects include the potential direct effects against cancer cells (André et al., 2017), the stimulation of the anticancer properties of the immune system and re-induction of tumor dormancy (Pasquier et al., 2007, 2010; André and Pasquier, 2009; André et al., 2013, 2017).

We designed a metronomic regimen relying mainly on oral medications and used a continuous LD methotrexate and cyclophosphamide backbone published in Colleoni et al. (2002). Besides, since MC may be given for a long period of time resulting in high accumulated doses of drugs received we wanted to avoid etoposide and temozolomide that have been previously reported to potentially induce secondary leukemia (Le Deley et al., 2005; Dufour et al., 2008; André et al., 2011). Both drugs are used in the most efficient pediatric metronomic regimen published so far (Kieran et al., 2005; Stempak et al., 2006; Sterba et al., 2006; Choi et al., 2008; Göbel et al., 2014; Robison et al., 2014; Ali and El-Sayed, 2016; Berthold et al., 2017). Vinblastine was also part of this regimen since this drug has limited hematological toxicity and anti-tubulin agents are known to have potent anti-angiogenic properties (Pasquier et al., 2007; Heng et al., 2016). Moreover, vinblastine can activate dendritic cells (André and Pasquier, 2009) and hence can be included in metronomic protocols (Pasquier et al., 2010). Celecoxib has been part of most pediatric metronomic regimen (Kieran et al., 2005; Sterba et al., 2006; Pasquier et al., 2007; Choi et al., 2008; Berthold et al., 2017) as it adds potential anti-angiogenic effects and tumor sensitization to chemotherapy (Stempak et al., 2006) and also display immune-modulating properties (Göbel et al., 2014).

The anti-tumor efficacy of this regimen seems predominant in LGG where two PRs were observed and 6 SDs among 10 evaluable patients, of which 7 patients continued treatment beyond 1 year and 2 patients had 2 years of treatment with ongoing stabilization. It should be emphasized that 9 out of evaluable 10 patients had previously been treated with vinblastine, which in most patients did not prevent a relapse or refractory disease at that time since the median duration of previous vinblastine treatment was only 4.4 months. Although we cannot rule out that the tumor stabilization was caused by a re-challenge by vinblastine, it seems likely that the addition of the other three compounds had an additive or synergistic effect. It should be emphasized that the patients with LGG in our series tolerated well the MC with >90% of dose intensity of vinblastine received during the first year of treatment but the dose of vinblastine is twice lower than the one used by Bouffet et al. (2012). Stempak et al. (2006) reported protracted stable disease in a patient with low grade astrocytoma using vinblastine and celecoxib.

When comparing the observed PFS of 70% at 2 years to other vinblastine-containing regimens for LGG, it can be noted that this PFS is similar or even slightly better as compared to the phase II with vinblastine monotherapy (Bouffet et al., 2012; Lassaletta et al., 2016) where 2-year PFS was ±60%. It should be emphasized that in this cohort of 51 patients there were 23% of NF1 patients that responded better to vinblastine as compared to non-NF1 patients (5-year PFS of 42%) (Lassaletta et al., 2016). In our series only one patient had NF1. Moreover, vinblastine was used as first line in the series as reported by Lassaletta et al. (2016), whereas our patients with LGG in majority had had ≥3 lines of chemotherapy. It should be stressed that in the Bouffet series 1 CR and 9 PRs were observed in 51 patients, which is a comparable response rate as in our series (2/10 patients with a PR). We previously reported a case-series of 4 patients with optic-pathway glioma that received vinblastine maintenance therapy after an induction with irinotecan and bevacizumab (IB). One patient responded to vinblastine after being stable after IB, while the other 3 patients remained stable, all of them having had 12 months of vinblastine therapy with a median follow-up of now more than 30 months (Heng et al., 2016). Our results in LGG are comparable in terms of PFS to the series of 12 LGG patients as described by Robison et al. (2014) using a MC regimen with continuous celecoxib, fenofibrate, thalidomide and alternating oral etoposide with cyclophosphamide. They observed 4 PR, 5 SD, and 3 PD with however only 7 patients reaching the 27 weeks of MC where in our series 7/10 had at least 1 year of MC. Elsewhere, Zapletalova et al. (2012) observed disease stabilization in 9/10 low grade tumors, among which LGG, using the COMBAT II regimen containing celecoxib, fenofibrate, vitamin D with alternating etoposide and temozolomide. No PFS was mentioned specifically for the patients with LGG.

The concept of MC was previously explored by several teams in non-brain and brain tumors. Two randomized studies were published that were performed in cohorts of pediatric patients with relapsed extra-cranial solid tumors. Pramanik et al. (2017) compared the effect of MC on PFS in pediatric patients progressing after at least two lines of chemotherapy. The MC regimen consisted of daily celecoxib and thalidomide with alternating periods of etoposide and cyclophosphamide, whereas the other arm received placebo. They showed no benefit on PFS for the entire group although subgroups of patients with none-bone sarcoma seemed to have some benefit (Pramanik et al., 2017). Senerchia et al. (2017) showed no benefit of 72 weeks of MC containing oral daily cyclophosphamide and twice weekly oral methotrexate after a conventional backbone of chemotherapy for localized osteosarcoma.

Several phase II trials with MC have been published in pediatrics (Kieran et al., 2005; Stempak et al., 2006; Sterba et al., 2006; Pasquier et al., 2007; Choi et al., 2008; Senerchia et al., 2017). Choi et al. (2008) used a MC regimen based on alternating oral etoposide, temozolomide, and cyclophosphamide in combination with either retinoic acid or celecoxib. The patient population consisted of high risk patients with several malignant histologies and predominantly residual disease at time of inclusion. One PR and four prolonged SDs were observed while 3 patients remained in remission after having no residual disease. The responding or stabilized patients had 6–12 months of MC (Choi et al., 2008). The responding patient had an oligodendroglioma. In our series one patient with anaplastic oligodendroglioma had SD for 2 years and still is progression-free. We acknowledge the fact that these are only two cases but may merit additional explorations given the limited therapeutic options for these patients at relapse. Kieran et al. (2005) explored a similar regimen with interesting responses in neuroblastoma, LGG, malignant fibrous histiocytoma, and medulloblastoma.

In our phase II clinical trial, no significant activity was observed in other brain tumor histologies but LGG. Nearly all patients with HGG (one excluded), ependymoma, medulloblastoma, or other BT were not stabilized by our regimen. This is in contrast with the series reported by Kieran et al. (2005) where prolonged remissions/stabilizations were observed in ependymoma (*n* = 4/5), medulloblastoma (*n* = 1/1) and optic pathway glioma (*n* = 1/1), after using a metronomic regimen with thalidomide, celecoxib and alternating etoposide and cyclophosphamide (Kieran et al., 2005). In the 19 patients with ependymoma reported by Robison et al. (2014), 2 PR, 10 SD, and 7 PD were observed with 37% having at least 27 weeks of MC. These observations were one of the rationale to explore a separate group of ependymoma in our phase II trial. The MC regimen consisted of alternating oral etoposide and cyclophosphamide with continuous thalidomide and celecoxib. We can consider that the lack of etoposide and/or temozolomide in our MC regimen led to an inadequate efficacy in our groups of BT (LGG excluded).

We want to emphasize that even if MC is considered to induce lower toxicity, that our series showed acceptable though notable AEs with a majority of grade 2 or 3 events and few grade 4 events. We had more grade 3/4 hematologic toxicity as compared to the series published by Ali and El-Sayed (2016) who used a similar MC scheme in combination with focal RT. A high clinical benefit rate of 76% was observed with only grade I hematological toxicity observed. It should be emphasized that pathologies and previous treatments were not comparable to our series.

## Conclusion

The four-drug regimen we report on here was rather well-tolerated and showed anti-tumor activity in relapsed LGG, even in patients having had previously received vinblastine treatment. These results warrant additional explorations in larger patients’ series while comparing to vinblastine mono-therapy.

## Author Contributions

AV was the principal investigator. AV, M-AH-M, RT, EJ, and NA contributed to trial design and methodology and data-analysis. SH was responsible for drug delivery and accountability. PD-L was responsible for central radiology review. AV, NA, PL, IA, PC, J-CG, NC, NS, and NE-W were responsible for including and treating patients and for source documents.

## Conflict of Interest Statement

The authors declare that the research was conducted in the absence of any commercial or financial relationships that could be construed as a potential conflict of interest.
